# Improving Risk Documentation in Laparoscopic Cholecystectomy: A Single-Centre Before-and-After Study of a Pre-printed Consent Form

**DOI:** 10.7759/cureus.95463

**Published:** 2025-10-26

**Authors:** Kapil Agrawal, Jaspreet Singh Kaur, Blanca Carioni, Sima Patel, Shumaila Tanveer, Sanaa Elgaddal

**Affiliations:** 1 Surgery, New Cross Hospital, Wolverhampton, GBR; 2 General Surgery, The Royal Wolverhampton NHS Trust, Wolverhampton, GBR; 3 General Surgery, New Cross Hospital, Wolverhampton, GBR; 4 General and Colorectal Surgery, New Cross Hospital, Wolverhampton, GBR

**Keywords:** consent form completion, conventional laparoscopic cholecystectomy, ethical and legal principles in medical practice, laparoscopic surgery, medical ethics, patient consent, patient safety culture, pre-printed consent, surgical informed consent, valid informed consent

## Abstract

Introduction: Informed consent is essential in surgery, requiring patients to receive clear information about risks, benefits, and alternatives. For laparoscopic cholecystectomy, the most commonly performed general surgical procedure in the UK, studies highlight inconsistencies in risk disclosure, with frequent omission of uncommon but serious complications. Such omissions may compromise patient understanding, shared decision-making, and medico-legal protection. This study aimed to assess whether a pre-printed, procedure-specific consent form could improve compliance with risk documentation.

Methods: We performed a retrospective, single-centre, before-and-after study at a UK district general hospital. Consecutive adult patients undergoing laparoscopic cholecystectomy were included across two study periods: baseline (December 2023-June 2024, *n* = 46) and post-intervention (November-December 2024, *n* = 23). Consent forms were reviewed against a predefined set of nationally recommended risks, derived from guidance and local consensus. The intervention consisted of a pre-printed consent form that listed these risks in a structured checklist format, with space for additional notes. The primary outcome was complete documentation of all predefined risks. Informal feedback from surgeons was also collected and thematically summarised.

Results: At baseline, none of the 46 forms achieved full compliance (0%). While common risks such as bleeding, infection, and conversion to open surgery were documented universally, other important risks were inconsistently recorded: bile duct injury (95.7%), bile leak (95.7%), adhesional obstruction (15.2%), chest infection (6.5%), and death (2.2%). Following the introduction of the pre-printed form, all 23 forms achieved complete compliance (100%). Surgeons described the new form as time-saving, clear, comprehensive, and helpful in enhancing patient trust.

Conclusion: The introduction of a pre-printed consent form significantly transformed risk documentation for laparoscopic cholecystectomy, increasing from 0% to 100%. This simple, low-cost intervention standardises practice, strengthens medico-legal robustness, and supports shared decision-making. Wider adoption across surgical services may improve consent quality and patient safety.

## Introduction

Informed consent is a cornerstone of modern surgical practice, ensuring patients receive information about the risks, benefits, and alternatives of treatment. Within the United Kingdom (UK), the General Medical Council (GMC) requires doctors to disclose material risks in a way that supports shared decision-making [1]. The Royal College of Surgeons of England provides similar guidance, emphasising that valid consent must include a clear discussion of risks, benefits, and reasonable alternatives [2].

The legal precedent set in Montgomery v Lanarkshire Health Board [3] reinforced this standard, establishing that clinicians must disclose any risk considered significant by a reasonable patient in the same situation. The Mental Capacity Act 2005 [4] further requires that patients are presumed to have capacity unless proven otherwise, and capacity must be assessed in a decision-specific and time-specific manner. For valid consent, patients must understand, retain, weigh, and communicate the relevant information.

Despite this framework, studies have shown substantial variability in consent documentation across surgical specialties [5-7]. In laparoscopic cholecystectomy, the most common general surgical procedure, serious but less frequent risks such as bile duct injury, adhesional obstruction, or death are often omitted [6]. Such omissions may compromise patient trust, impair shared decision-making, and weaken medico-legal protection [7-9].

Handwritten consent forms introduce additional challenges, including illegibility, abbreviations, and omissions due to human error. Pre-printed, procedure-specific consent forms provide a structured solution, standardising the process and reducing variability [10-12]. Evidence from other surgical fields demonstrates improved documentation and patient understanding when pre-printed forms are used [11,12].

At our institution, an internal review revealed omissions in risk documentation during consent for laparoscopic cholecystectomy. We therefore developed and introduced a pre-printed consent form to support surgeons in achieving comprehensive, standardised risk disclosure. This study aimed to evaluate the impact of the intervention on documentation compliance.

## Materials and methods

This was a retrospective, single-centre, before-and-after study conducted in the Department of General Surgery at a UK district general hospital. Consecutive adult patients (≥16 years) undergoing laparoscopic cholecystectomy were included during two study periods: baseline (December 2023 to June 2024, n = 46) and post-intervention (November to December 2024, n = 23). Patients lacking capacity, those requiring parental or alternative consent, and cases with missing consent forms were excluded.

Consent forms were reviewed against a predefined set of procedure-specific risks derived from national guidance and local clinical consensus. The risk set included bleeding, infection, bile duct injury, bile leak, injury to other organs, conversion to open surgery, deep vein thrombosis/pulmonary embolism (DVT/PE), hernia, intra-abdominal collection, adhesional obstruction, keloid formation, chest infection, failure to relieve symptoms, and death. Complete compliance was defined as documentation of all these risks.

The intervention was a pre-printed laparoscopic cholecystectomy consent form, designed in collaboration with consultant surgeons and clinical governance staff. The form listed the agreed risks in a checklist format with tick boxes and free-text space for additional notes. It also prompted clinicians to offer a copy of the form to patients. The form was approved for departmental use in October 2024 and introduced through ward and theatre distribution as well as teaching sessions for surgical staff.

The primary outcome was the proportion of consent forms with complete risk documentation. Two investigators independently reviewed each form, with discrepancies resolved by consensus. Informal feedback on the new form was also collected from surgeons most frequently responsible for taking consent. The feedback was summarised thematically.

## Results

Baseline documentation

**Figure 1:**
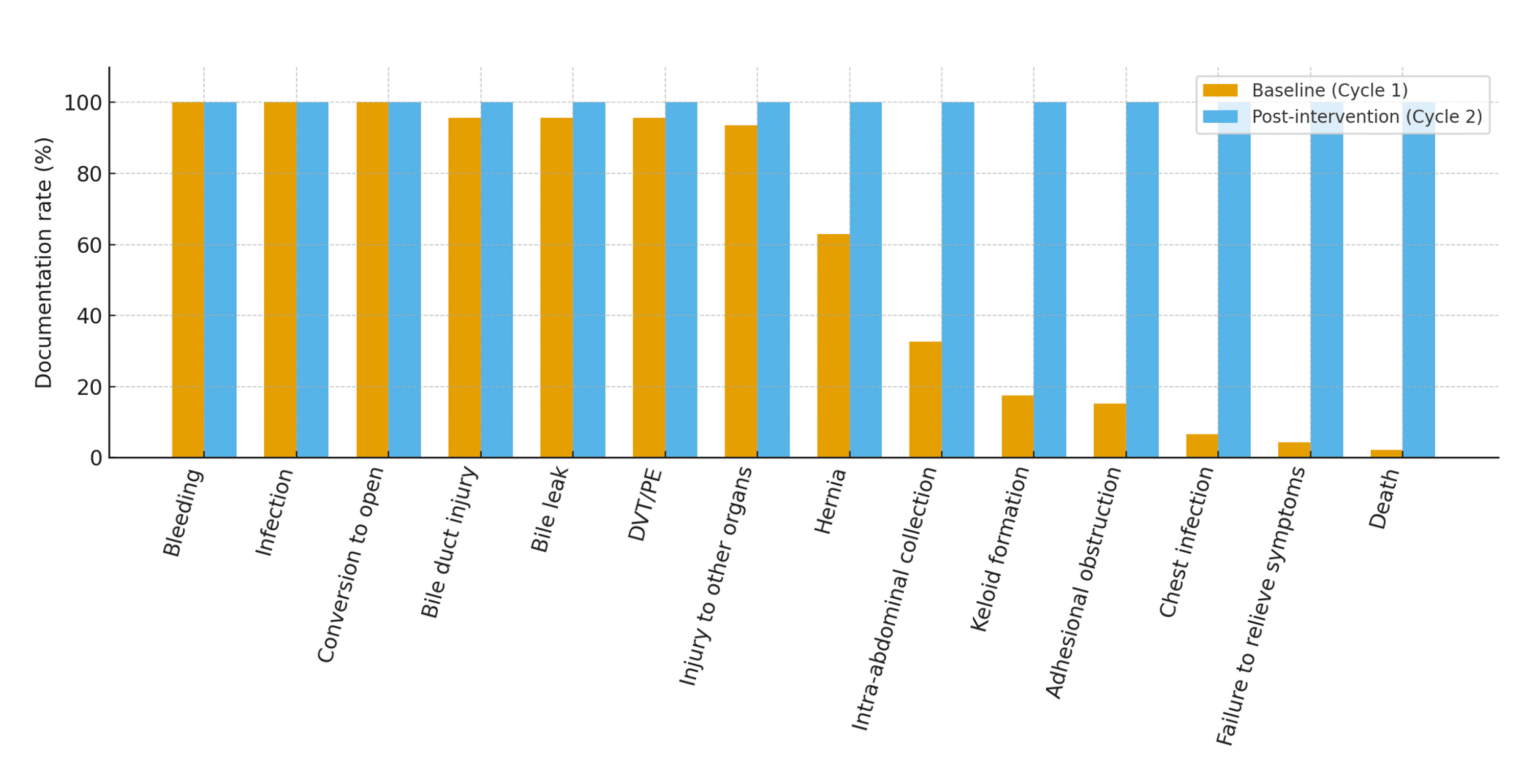
Risk documentation rates before-and-after intervention. DVT = deep vein thrombosis; PE = pulmonary embolism; Baseline period = December 2023–June 2024 (n=46); Post-intervention period = November–December 2024 (n=23).

During the baseline period (December 2023 to June 2024), 46 consent forms for laparoscopic cholecystectomies were reviewed. None of the forms demonstrated complete compliance with the predefined risk set. While bleeding, infection, and conversion to open surgery were universally documented, several important complications were inconsistently included. Bile duct injury and bile leak were present in 44 of 46 cases (95.7%), whereas adhesional obstruction was noted in seven of 46 cases (15.2%), keloid formation in eight of 46 cases (17.4%), chest infection in three of 46 cases (6.5%), failure to relieve symptoms in two of 46 cases (4.3%), and death in one of 46 cases (2.2%) were rarely documented. Full documentation rates are provided in Table 1, and the comparison of baseline versus post-intervention documentation across all risks is shown in Figure 1.

**Table 1:** Documentation of risks before-and-after intervention DVT = deep vein thrombosis; PE = pulmonary embolism; Baseline period = December 2023–June 2024 (n=46); Post-intervention period = November–December 2024 (n=23).

Risk	Baseline (n=46)	Post-intervention (n=23)
Bleeding	46 (100%)	23 (100%)
Infection	46 (100%)	23 (100%)
Conversion to open	46 (100%)	23 (100%)
Bile duct injury	44 (95.7%)	23 (100%)
Bile leak	44 (95.7%)	23 (100%)
DVT/PE	44 (95.7%)	23 (100%)
Injury to other organs	43 (93.5%)	23 (100%)
Hernia	29 (63.0%)	23 (100%)
Intra-abdominal collection	15 (32.6%)	23 (100%)
Keloid formation	8 (17.4%)	23 (100%)
Adhesional obstruction	7 (15.2%)	23 (100%)
Chest infection	3 (6.5%)	23 (100%)
Failure to relieve symptoms	2 (4.3%)	23 (100%)
Death	1 (2.2%)	23 (100%)

Post-intervention documentation

**Figure 2:**
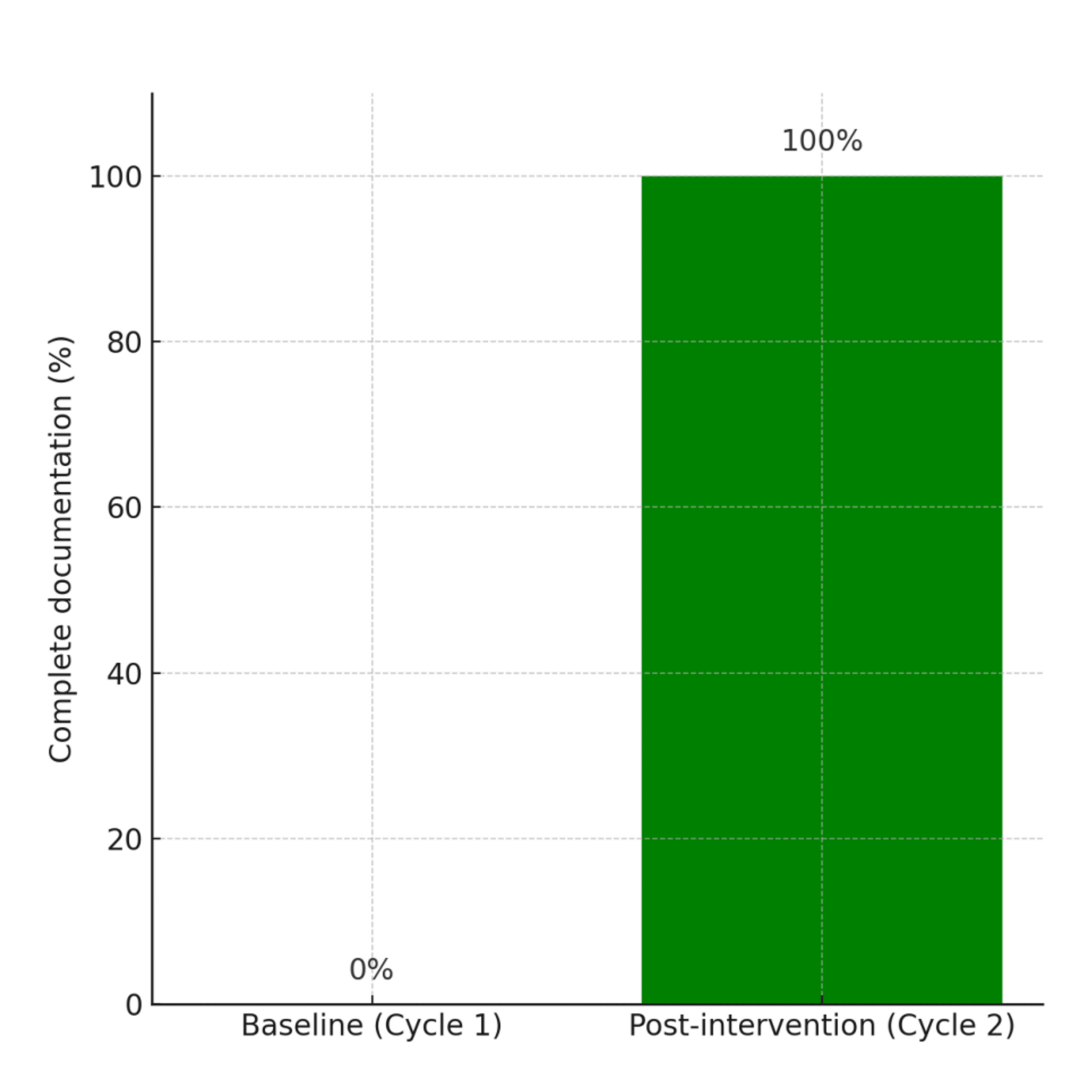
Overall compliance improvement. DVT = deep vein thrombosis; PE = pulmonary embolism; Baseline period = December 2023–June 2024 (n=46); Post-intervention period = November–December 2024 (n=23).

Following the introduction of the pre-printed consent form (November-December 2024), 23 consent forms were reviewed. All forms demonstrated complete compliance, with every recommended risk documented in 23 of 23 cases (100%). This represents a dramatic improvement from baseline, where no form achieved full compliance. The overall change in compliance from 0% (0/46) to 100% (23/23) is illustrated in Figure 2.

Surgeons' feedback

**Figure 3:**
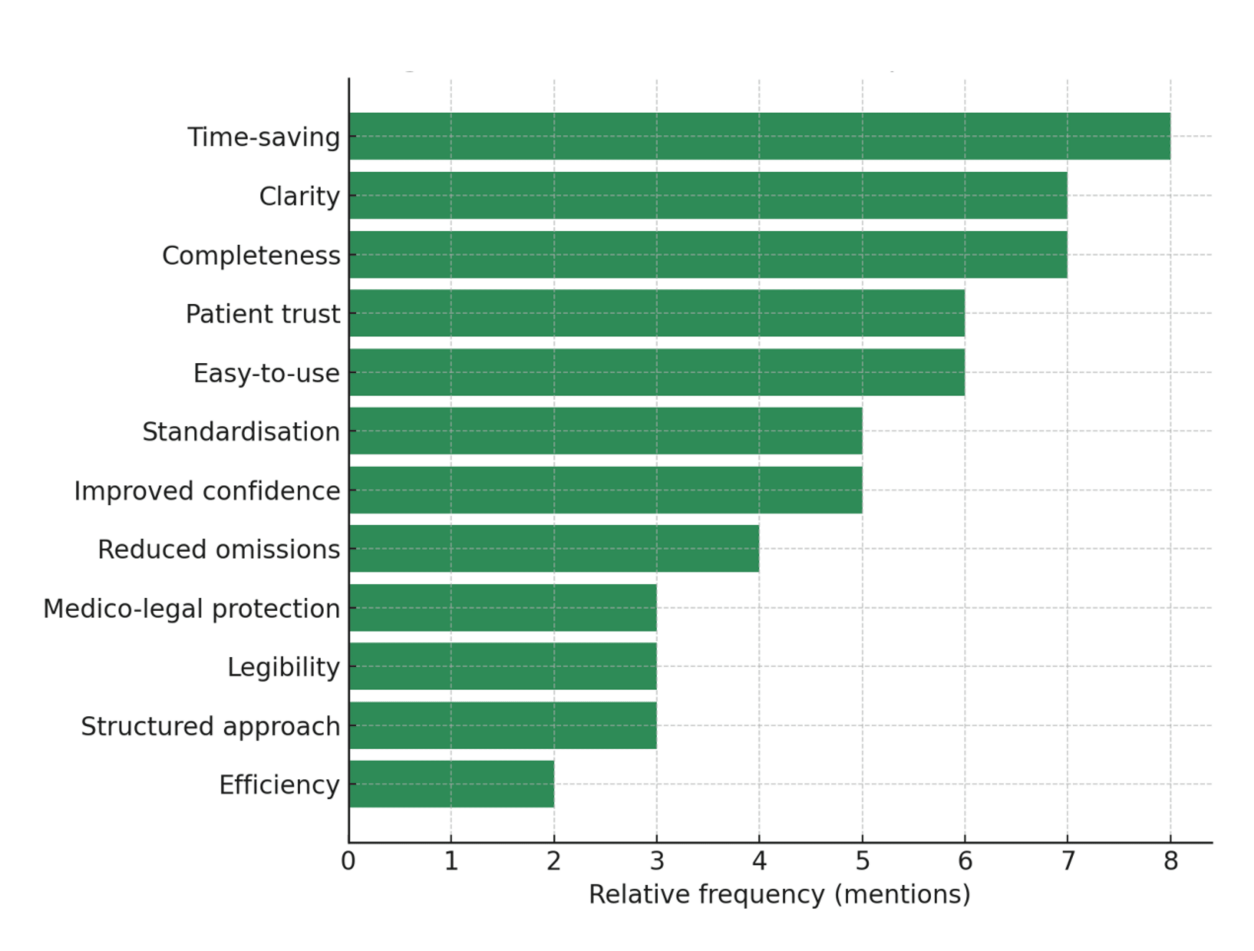
Surgeons' feedback themes on pre-printed consent form.

Surgeons provided informal feedback on the new form, highlighting several recurring themes. They reported that the form saved time, reduced reliance on memory, and eliminated omissions. The structured checklist was seen as improving both legibility and clarity while providing reassurance about medico-legal robustness. Importantly, surgeons also felt that the form improved patient trust by demonstrating thoroughness and consistency. These themes are summarised in Figure 3.

Pre-printed consent form

**Figure 4:**
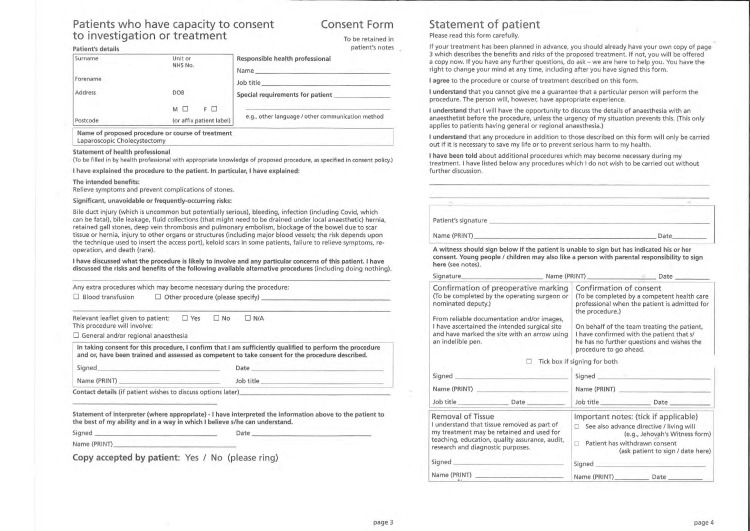
Pre-printed laparoscopic cholecystectomy consent form.

The intervention itself is shown in Figure 4, which depicts the pre-printed laparoscopic cholecystectomy consent form developed for this study. The template provides a structured checklist of risks with tick boxes, alongside space for free-text notes to allow documentation of patient-specific considerations.

## Discussion

This study demonstrates that introducing a pre-printed, procedure-specific consent form for laparoscopic cholecystectomy significantly improved the completeness of risk documentation, increasing it from 0% to 100%. At baseline, common complications such as bleeding, infection, and conversion to open surgery were universally documented, yet other important risks, including death, adhesional obstruction, or failure to relieve symptoms, were rarely recorded. Following the introduction of the pre-printed form, all risks were consistently included, eliminating omissions and standardising practice across clinicians.

Comparison with existing literature

Our baseline findings are consistent with prior studies, which have demonstrated variability and incompleteness in surgical consent. Courtney and Royle reported inconsistent use of procedure-specific forms for laparoscopic cholecystectomy, with frequent omissions of key risks [5]. Sebastian et al. similarly found wide variation in consent documentation across UK surgical practice, raising concerns about both patient autonomy and medico-legal protection [6]. Uzzaman et al. also noted that consenting practice for laparoscopic cholecystectomy often failed to include significant complications such as bile duct injury [7].

Evidence from other specialties has suggested that structured, procedure-specific consent forms improve documentation. Anderson and Wearne highlighted the importance of completeness and clarity in elective surgical consent [8]. Chan et al. demonstrated the legal and practical implications of the Montgomery judgement, underscoring the clinician's duty to disclose all material risks [9]. Taylor et al. described how Plan-Do-Study-Act (PDSA) cycles can be used to drive sustainable quality improvement in healthcare [10]. St John et al. demonstrated that electronic procedure-specific forms improve legibility and reduce omissions [11]. Baker et al. found similarly positive outcomes with pre-printed forms in ophthalmic surgery [12]. Sivanadarajah et al. confirmed that the readability of consent documents is a frequent challenge in surgical practice [13]. Beyond consent specifically, structured safety tools such as the WHO surgical safety checklist have been shown to reduce variability, improve communication, and enhance patient outcomes, reinforcing the principle that checklists can drive safer, more standardised care [14]. Importantly, the intervention aligns local practice with national recommendations for gallstone disease management, as outlined by the National Institute for Health and Care Excellence (NICE) and the European Association for the Study of the Liver (EASL) [15,16]. Our study supports and extends these findings, showing that a simple intervention can achieve complete compliance in a high-volume general surgical procedure.

Implications for clinical practice

The implications of this improvement are multifaceted. First, by eliminating omissions, the pre-printed form ensures patients receive consistent information about both common and rare complications. This strengthens shared decision-making, allowing patients to balance risks and benefits in an informed manner. Second, the intervention supports clinicians, particularly trainees, by reducing reliance on recall and providing cognitive prompts. Third, medico-legal robustness is enhanced, as documentation reflects a complete discussion of material risks, aligning with the principles established in Montgomery v Lanarkshire Health Board [3].

Feedback from surgeons suggested that the form saved time, reduced stress when obtaining consent, and improved patient trust. These benefits align with broader evidence that structured communication tools improve patient satisfaction and confidence in the consent process [17-19]. In addition, the improved legibility and elimination of abbreviations address another frequent weakness of handwritten consent forms, which can undermine patient comprehension [13].

Beyond these immediate benefits, recent studies further expand this evidence base. Shirley et al. reported similar quality improvements using standardised consent templates in surgical audit frameworks [20]. Gardiner et al. highlighted that effective consent is a shared, multidisciplinary process that emphasises nursing and allied health involvement [21]. Pittalis et al. reviewed informed consent practices in sub-Saharan Africa, identifying structural and educational barriers that mirror those in high-income settings [22]. Gebrehiwot et al. demonstrated that patients’ perceptions of consent quality strongly correlate with clarity and completeness of risk explanation [23]. Similarly, Gardiner et al. and Kumru et al. reported that patients value structured communication and written materials that reinforce understanding [24,25]. Vieira et al. found variability in consent standards even within European teaching hospitals, calling for formal training and institutional templates [26]. Systematic reviews by Schenker et al. and Dwamena et al. confirmed that interventions to improve consent comprehension and promote patient-centred communication significantly enhance understanding and satisfaction [27,28]. Hoffman et al. underscored the link between evidence-based medicine and shared decision-making, while Hall et al. emphasised that informed consent remains a clinical process grounded in ethical communication [29,30].

Strengths and limitations

The key strength of this study is the magnitude and clarity of its findings: a simple, low-cost intervention achieved complete and immediate improvement. The before-and-after design, with consecutive case inclusion, strengthens internal validity. Importantly, the intervention has been embedded into routine practice, demonstrating sustainability beyond the re-audit period.

However, several limitations should be acknowledged. This was a single-centre study, which may limit generalisability. The post-intervention sample size was modest, reflecting the relatively short re-measurement period. Patient-reported outcomes, such as comprehension, satisfaction, or trust, were not formally assessed. While surgeon feedback suggested perceived patient benefit, this remains an important area for future work. The feedback itself was collected informally rather than through a structured survey, which limited the depth of analysis. Finally, the study did not assess potential balancing measures, such as additional time burden or cost, although informal reports suggested that the intervention saved time rather than added to it.

Future directions

Several avenues for further research emerge. First, patient perspectives should be incorporated into future evaluations, as ultimately the success of consent interventions should be judged by whether patients feel adequately informed and engaged. Second, integration into electronic consent platforms may further enhance sustainability, reduce paperwork, and improve accessibility of documentation. Third, wider adoption and evaluation across multiple centres and surgical specialties would help determine generalisability. High-volume procedures, such as hernia repair, appendicectomy, and colectomy, may particularly benefit from standardised templates. Finally, longitudinal follow-up could assess whether improvements are sustained over the years and whether medico-legal claims related to consent are reduced.

Overall significance

Informed consent remains a cornerstone of ethical surgical practice, yet variation in quality persists despite clear professional and legal frameworks. By introducing a structured, procedure-specific template, our study demonstrates that consent quality can be reliably standardised. The intervention is simple, scalable, and cost-free beyond printing, making it attractive for wider adoption. By supporting clinicians and empowering patients, these tools can help realise the vision of consent as a truly collaborative process rather than a perfunctory formality.

## Conclusions

The introduction of a pre-printed, procedure-specific consent form for laparoscopic cholecystectomy transformed documentation compliance from 0% to 100% at our institution. By embedding a structured checklist into routine practice, the form eliminated omissions, standardised risk disclosure, and enhanced both patient safety and medico-legal protection. Surgeons found it time-efficient and easy to use, with clear benefits for patient trust and departmental consistency.

Although this was a single-centre study, the intervention is simple, low-cost, and readily scalable. Wider adoption across surgical services could significantly strengthen the quality of consent, particularly in high-volume procedures. Incorporating patient feedback and exploring integration into electronic consent platforms represent important next steps. In the long term, such measures have the potential to make the consent process more reliable, transparent, and patient-centred.
